# Validation and Adjustment of the Patient Experience Questionnaire (PEQ): A Regional Hospital Study in Norway

**DOI:** 10.3390/ijerph18137141

**Published:** 2021-07-03

**Authors:** Seth Ayisi Addo, Reidar Johan Mykletun, Espen Olsen

**Affiliations:** Department of Innovation, Leadership and Marketing, University of Stavanger Business School, University of Stavanger, 4036 Stavanger, Norway; reidar.j.mykletun@uis.no (R.J.M.); espen.olsen@uis.no (E.O.)

**Keywords:** patient experiences, PREMs, psychometrics, CFA, hospital, second-order factor, Norway

## Abstract

This paper assesses the psychometric qualities of the Patient Experience Questionnaire (PEQ), thereby validating a patient-oriented measurement model in a hospital environment, and modifies the model based on empirical results. This study employed survey data gathered by the Norwegian Institute of Public Health from adult inpatients at somatic hospitals in the Health South-East RHF in Norway. The survey engaged 4603 patients out of 8381 from five main hospitals in the region. The study found that an eight-factor model of the PEQ generally showed good fitness to the data, but assessment of discriminant validity showed that this was not the optimal factor solution among four of the eight dimensions. After comparing models, the study proposed a model with a second-order factor for four of the factors: “nurse services”, “doctor services”, “information”, and “organization”, collectively named “treatment services”. The proposed model demonstrated good validity and reliability results. The results present theoretical and practical implications. The study recommends that inferential analyses on the PEQ should be done with the second-order factor. Furthermore, a revision of the PEQ is recommended subject to more confirmatory studies with larger samples in different regions. The study indicates a second-order factor structure for assessing and understanding patient experiences—a finding which has both theoretical and managerial implications.

## 1. Introduction

Healthcare professionals are facing heavy pressure to meet the growing needs of patients such as medical, physical, and psychological healthcare needs [1] as well as patients’ expectations of quality services, products, and performance [2]. This is due to the increasing and alarming rate of morbidity and multi-morbidity in Western countries [3], together with aging populations and the healthcare needs of the aged. Pressure on healthcare professionals has increased in recent times with the outbreak of global pandemics such as COVID-19. Notwithstanding these morbidity rates and the growing needs of patients, healthcare providers and professionals are expected to ensure positive patient experiences. This study, focusing on hospitals and their professionals, seeks to examine patients’ experiences with hospital service climates, focusing on the psychometric quality of a patient-reported experience measure (PREM).

The endeavour of gathering patients’ experiences with healthcare has gained popularity, thus resulting in the development of PREMs that have been used in surveys in various countries [4,5,6,7,8]. In a bid to clarify the meaning of patient experiences, Wolf and Jason [9] synthesized various definitions of the concept and maintained that patient experiences comprise individual as well as collective events and occurrences that happen in the process of caregiving, and this has strong links with patients’ expectations and how they were met. Wagland et al. [10] noted that significant progress has been made in understanding patient experience. The concept is viewed as interactions of patients with aspects of the healthcare delivery such as nurse services, doctor services, organization of the caregiving process in hospitals, and information delivery, where these aspects (dimensions) culminate in the entire continuum of experience that patients have with healthcare, as reported by the patients. 

From patients’ perspectives, interactions with dimensions of healthcare have been theoretically underpinned by the Donabedian framework for assessing healthcare quality [11], which is considered the most widely used in the healthcare sector to assess quality [12]. According to this framework, quality of healthcare can be assessed by making inferences under three categories: structure, process, and outcome. The structure deals with the setting in which care is given, for instance, facilities, equipment, and human resources. The process deals with what is done in giving and receiving care, for instance, nurse and doctor services as well as good communication and information sharing between patients and hospitals; and lastly, the outcome deals with the effects of care on health and well-being [11,13].

Increased understanding of patient experiences of hospital climate has similarly been aided by increased research and several studies on measuring the construct. Measurements in social science provide adequate guidelines for assessing phenomena and people’s attributes that are not directly and easily observable [14]. Employing poor and inadequate measures in research can be very costly to practice, in terms of drawing invalid conclusions, making policy decisions based on false information, and wasting respondents’ time and efforts [15]. DeVellis [15], however, indicated that a major challenge to developing adequate measures in social science is the immaterial nature of social science constructs supported by constantly changing theories. This makes measurements in social science susceptible to constant changes in performance and adequacy in assessing the constructs. Consequently, social science measures need to be constantly reviewed and reassessed to keep them abreast with changing theories and constructs and to uphold their validity and reliability. Therefore, reassessing PREMs to ensure adequate psychometric qualities is essential for theoretical and practical advancement of knowledge of patients’ experiences, hence the focus and aim of this study.

### Justification of the Study

The goal to accurately measure patient experiences has resulted in several PREMs for general and specialized healthcare [5]. The questions and dimensions that these PREMS have produced are indicative of patients’ shared experiences. Most of these measures identified similar dimensions of experiences, such as those relating to nurse services, doctor services, information and communication, hospital organization and standards, and discharge from the hospital [5,6,16,17,18]. Although some of these studies differed with regard to the naming of the dimensions, the content of the items remained very similar among the PREMs. This study is underpinned by two main justifications: (i) psychometric statistical analyses have evolved over the years with more robust tools in validating scales; and (ii) due to the plethora of patient experience measures and unascertained psychometric qualities, existing PREMs should be re-examined to ascertain their validity and reliability, rather than developing new ones. These justifications are elaborated below.

The Norwegian Institute of Public Health (NIPH) conducted a survey in the east health region among a few hospitals, adapting an earlier validated PREM, the Patient Experience Questionnaire (PEQ) [8]. In the development and validation study, Pettersen, Veenstra (8) employed literature reviews, focus groups, pilot studies, and two cross-sectional surveys (1996 and 1998) across 14 hospitals in Norway. The study used exploratory factor analysis, a reliability test (Cronbach’s alpha), and a construct validity test. The study found 10 factors and 20 final items out of an initial 35 items: “information on future complaints”, “nursing services”, “communication”, “information examinations”, “contact with next-of-kin”, “doctor services”, “hospital and equipment”, “information medication”, “organization”, and “general satisfaction”. All the factors recorded Cronbach’s alpha scores between 0.61 and 0.83. Construct validity was also ascertained in the study by examining the relationship between the instrument and demographic factors such as age and gender. Stressing the lack of valid and reliable instruments, Pettersen et al. [8] concluded that it is imperative to re-examine existing patient experience measures so as to improve methodology. They further recommended employment of the PEQ for future in-patient experience surveys, hence the choice for the current study. Although this measure was adapted and modified for use by the NIPH, the performance of the measure should be called into question because this measure was developed and validated more than a decade ago. Psychometric analyses are evolving with more robust validating tools and methods, and this is evident in the study by Pettersen et al. [8] where issues such as discriminant validity and measurement invariance as well as other psychometric issues were absent in the analyses—a gap that the current study tackles.

Beattie et al. [19] also noted the problem of multiple patient experience measures with unascertained psychometric quality. This problem has hindered the use of data from patient experience surveys to adequately improve and sustain quality of care in hospitals. In the systematic review, Beattie et al. [19] developed a matrix to help choose PREMs for research and to identify research gaps in existing ones. This matrix showed that the PEQ study by Pettersen et al. [8] lacked analyses such as criterion-related validity. On this basis, the current study asserts that rather than developing more PREMs (which seem already saturated), existing ones should be re-examined, as recommended earlier by Pettersen et al. [8], in light of current analyses and conceptual underpinnings. This need for re-examination has also been recommended by other systematic reviews on patient experience [20,21].

Additionally, some PREMs have been developed in Norway to capture the phenomenon of patient experiences with general health practice as well as experiences with specific health issues and fields, with most of them asking questions on general patient satisfaction [8,18,22,23]. Haugum et al. [20] similarly recommended the need to repeat patient experience surveys and their outcomes in order to generate more validated instruments, as they are potentially affected by contextual factors. By inference, it can be said that the underlying psychometric rigors of a PREM can dwindle as they are employed over a long period. Although several surveys exist on patient experiences on various issues [2,24,25,26,27], a re-analysis of the psychometric performance of any particular measure is lacking. The quest to improve healthcare delivery and hospital service climate based on patients’ experiences should begin with ascertaining the psychometric quality of PREMs. Based on these justifications, the purpose of this article is to test the psychometric qualities of the PEQ, thereby validating a measurement model in a hospital environment.

## 2. Materials and Methods

### 2.1. Sample and Data Collection

This study employed anonymous survey data from the Norwegian Institute of Public Health gathered from adult inpatients at somatic hospitals in the Health South-East RHF in Norway. These somatic hospitals dealt with issues generally affecting the bodies of patients and thus, were not specialized. The survey was started by the Norwegian Knowledge Centre for Health Services in the fall of 2015 and was continued at the Norwegian Institute of Public Health in the first quarter of 2016. It is worth noting that the last major reform and restructuring done in the Norwegian health sector was in 2002; where ownership of hospitals was transferred to the state. Thus, although changes have been made over the years since then, they are minor and incremental to the 2002 reform, focusing more on better standardization. These changes may therefore not affect this study in a major way. The survey engaged patients from 5 main hospitals in the region who were admitted for at least a day. The eligibility criteria were patients who were admitted between October and November in 2015 and who were admitted to the hospitals for at least one night. The study excluded outpatients. Patients who visited the 5 hospitals were identified through their contact information after they were discharged. Questionnaires were sent to their respective addresses via post mail with a return envelope. About 8381 patients were eligible and contacted. The total number of respondents who completed and returned their questionnaires was 4603, yielding a response rate of 54.92%. Patients were asked to consider various aspects of their experience being admitted. The questionnaire aimed at using feedback to identify which areas are working well and which areas the hospital should work to improve. 

### 2.2. Instrument

The Patient Experience Questionnaire (PEQ) comprised 8 dimensions and 33 items as well as items on patient safety, patient satisfaction, and overall health benefits and health level. The NIPH adapted the questions for the survey from the PEQ developed and validated by Pettersen et al. [8]: “nurse services” (items N1–N7), “doctor services” (items D1–D7), “information” (items IF1–IF3), “organization” (items OR1–OR4), “next of kin” (items NK1 and NK2), “standard” (items S1–S6), “discharge” (items DC1 and DC2), and “interaction” (items IT1 and IT2). These items were measured on a 5-point Likert scale ranging from “Not at all” (1) to “To a very large extent” (5). Patient safety was measured with 12 items, while patient satisfaction, health benefit, and health level were measured with 1 item each. Background information, such as questions on whether or not the patient chose the hospital they were admitted to, was also included in the questionnaire.

### 2.3. Data Analysis

#### 2.3.1. Preliminary Analyses

The study analysed the data with the aid of Microsoft Excel (Microsoft, Redmond, WA, USA), SPSS v.24 (IBM Corporation, Armonk, NY, USA), and AMOS v.25 (IBM Corporation, Armonk, NY, USA). Preliminary analysis (such as checking for normality, outliers, and missing value analysis) was conducted in SPSS. The missing values were found to be not at random, and therefore being mindful of how they were replaced was necessary. The study chose to use multiple imputations to replace them as recommended for non-randomness [28,29]. However, the 5 different imputations generated could not be pooled in AMOS as a single imputation for the estimation of the model. Thus, the missing values were eventually replaced with the series mean method. Analysis was performed mainly on the data with missing values due to their non-randomness and also due to the subject matter under investigation being patient experiences; as the study wanted to capture accurate measurements by the respondents. In order to ensure maximum privacy of respondents and still maintain relevant variables for analysis, departments for the analysis were aggregated into medical departments (Med) and surgical departments (Kir) across the hospitals based on the more specific and varied information on units in the hospitals provided by participants. This aggregation was performed according to the departmental codes for health institutions provided by the Norwegian Health Authority.

#### 2.3.2. Measurement Model Development

The initial measurement model (Model 1) was developed in AMOS without modification indices (due to the exclusion of missing values). Missing values were replaced with the series mean method after the estimation of the initial model to obtain modification indices for correlating error terms among the items and improving the fitness of the model (Model 2). It is noteworthy that the missing values were only replaced in order to generate a full estimation with modification indices for correlating the error terms. Although all subsequent models after the initial model were estimated with the correlated error terms, estimations were done on the data with missing values, with the aim of obtaining a more accurate fit of the data to the models.

The initial model with modifications (correlated error terms), Model 2, was compared with 6 other models (Models 3–8), obtained by combining some dimensions into a single factor to further justify the fitness of the modified initial model. These combinations were based on the correlation coefficients between the dimensions. In addition, a proposed model containing a second-order factor for “nurse services”, “doctor services”, “information”, and “organization” was also developed and compared with the initial modified model based on the validity tests, correlation analyses, and theoretical justifications (wording of questions). Fitness of all the models was ascertained using the following indices: Comparative Fit Index (CFI), Tucker Lewis Index (TLI), Root Mean Square Error of Approximation (RMSEA), and the PCLOSE. The thresholds recommended by Hu and Bentler [30] are presented in Table 1.

#### 2.3.3. Validity and Reliability

Validity in this study was ascertained using convergent, discriminant, and predictive validity tests. Convergent validity deals with the relationship between a latent construct (patient experience dimensions) and its items [31]. The average variance extracted (AVE) was used to check convergent validity, where values must be at least 0.50, indicating that at least half of the variance in the construct (dimension) is explained by its items. Discriminant validity focuses on a construct and its items in relation to other constructs—that is, how different one construct (or dimension) and its items are from other constructs in the model [31]. Discriminant validity was examined using the Fornell–Larcker procedure, where discriminant validity is supported when the square root of the AVEs is greater than the correlation coefficients between the constructs [32]. Predictive validity focuses on the ability of the measure and dimensions to relate to and predict previously ascertained outcomes in literature. This was determined through correlation and regression analyses between patient experiences (and dimensions) and outcome variables (patient satisfaction, health benefits, and health level) with the aid of SPSS. Reliability of the measurement model was also determined using composite reliability values for every dimension of the patient experience measure, with a recommended value of at least 0.70 to ascertain its repeatability in different contexts.

### 2.4. Ethical Considerations

This study, with regard to data collection, analysis, and compilation, was conducted within the ethical and legal provisions and guidelines of the Norwegian Institute for Public Health (NIPH) and the University of Stavanger. The Norwegian Data Protection Authority and the Norwegian Directorate of Health approved the procedures in the survey. The hospital data protection official assessed the data processing in the hospitals where survey extension took place. Informed consent was obtained from participants in the survey. Respondents were informed that participation was voluntary and they were assured of confidentiality of the information they will provide. Respondents were also informed that they could opt out of the survey at any point as well as the procedure for opting out if they wished. Data was stored in a safe repository with a password, only accessed by the researchers. This study did not present results that revealed patients’ identities, thus maintaining anonymity of respondents and confidentiality of responses. All relevant ethical requirements were duly upheld.

## 3. Results

### 3.1. Preliminary Analysis and Sample Characteristics

The study made use of responses from 4603 participants. Outliers were recorded for some of the questions, but this was to be expected considering the varied background characteristics, such as age and number of days spent in the hospital, which could influence participants’ experiences. Nonetheless, most of these outliers were not deemed extreme based on the 1.5 and 3.0 interquartile ranges. Normality was also ascertained, using the −2 and +2 range [33], for all items of patient experience, except the kurtosis value for one item on “nurse services” and one item on “doctor services”. Overall, the data could be said to be normally distributed to a large extent. The sample for the study was taken from five hospitals and characterized by a somewhat fair age distribution of patients across three groups: 60 years and below, between 61 and 73 years, and 74 years and above. Most of the respondents were admitted for three or fewer days, and more of them were also admitted to the medical department aggregate (Med). Table 2 presents the sample characteristics for this study.

### 3.2. Initial Measurement Model Development, Modifications, and Comparisons

The initial CFA model (Model 1, Table 3), with the eight dimensions of patient experience, was then developed to be tested. The model showed acceptable fitness to the data based on fitness indices (CFI = 0.91; TLI = 0.89; RMSEA = 0.06; PCLOSE = 0.00). Nonetheless, there was a need to improve the fitness through modifications in order to reduce measurement errors and to obtain more accurate loadings of the observed items on their dimensions. Some modifications were made by drawing covariance between some error terms on the same dimensions with the rationale that, by virtue of sharing commonalities on the dimension, they are more justified to share similar error terms, thus reducing duplications of random measurement error of items. In total, 19 modifications were made based on the covariance coefficients, with the highest coefficient as 895.667 between S2 and S4 (“standard”) and the lowest as 40.390 between D4 and D7 (“doctor services”). Aside from the coefficients, these modifications were theoretically justified. For example, the item D2 was worded, “Did you find that the doctors took care of you?”, and D4 was worded as “Did the doctors have time for you when you needed it?” Participants may have given closely related responses due to the phrases “taking care” and “having time when you needed”; therefore, it was no surprise that they shared similar error terms, leading to considerable covariance coefficient. These statistical and theoretical justifications were made for each covariance drawn. The most modifications were made to “doctor services” (seven), followed by “standard” (five), “nurse services” (four), “information” (two), and “organization” (one). No modifications were made to “next of kin”, “discharge”, or “interaction”, owing to very low covariance coefficients (below 20). The initial model with these modifications (Model 2 in Table 3) thus produced excellent fitness values for all indices. Furthermore, the model was compared with six other models (see Section 2), where the initial model with modifications showed the best fitness to the data. The fitness indices of the initial model before and after modifications, as well as those of the six alternative models for comparisons, are presented in Table 3.

### 3.3. Measurement Invariance across Hospital Departments Aggregated into Two Groups

Model 2 was further examined for invariance across three categories: configural, metric, and scalar. Measurement invariance tests seek to ascertain whether the measurement model differs across variant groups in a data. The goal is to achieve little or insignificant variance across these groups in order to inspire confidence in the ability of the measure to generate accurate responses and assessments across groups [34]. Configural invariance results (see Model 9, Table 3) showed that the model had acceptable-to-excellent fitness to the data, thus ascertaining configural invariance for the eight-factor patient experience measure across the two hospital department aggregates. With regard to metric invariance, the chi-squared test showed that the fully constrained model and the unconstrained mode were different across the department groups and, thus, not metrically invariant. However, MacKenzie et al. [35] maintained that “full metric invariance is not necessary for further tests of invariance and substantive analyses to be meaningful, provided that at least one item (other than the one fixed at unity to define the scale of each latent construct) is metrically invariant” (p. 325). Thus, the critical ratios test was performed to examine whether the dimensions and the items were metrically invariant enough for further meaningful analyses. The analysis revealed that for all dimensions, with the exception of “next of kin”, there was at least one item that was not statistically significant (metrically invariant) besides the item that was constrained for that dimension in the model. This means that the two items on the “next of kin” dimension had significantly different loadings (parameters) across the aggregated departments. Nonetheless, this test showed the model was metrically invariant across the departments to a large extent. The results of this test are presented as a supplementary table (Table S1). Scalar invariance was then examined for the model based on the differences in the measurement intercepts. The analyses showed that the model did not have scalar invariance. Differences in intercept estimates of items between the departments were computed, showing that almost all the items did not have scalar invariance across the two departments. The results are presented as a supplementary table (Table S2).

### 3.4. Reliability

Reliability for the measure was ascertained using composite reliability (CR) values. Generally, CR values above 0.70 are deemed acceptable to justify reliability. From Table 4, it is seen that all the dimensions recorded CR values above 0.70, with the highest being “doctor services” (0.92) and the lowest being “interaction” (0.72).

### 3.5. Convergent and Discriminant Validity

Convergent validity was examined using the AVE values, where an AVE value of at least 0.50 is considered acceptable [31]. Table 4 shows that all dimensions, with the exception of “standard”, recorded values above 0.50, thus ascertaining convergent validity. Discriminant validity was ascertained using the Fornell–Larcker procedure. There, discriminant validity is supported when the square root of the AVEs is greater than the correlation coefficients between the constructs [32]. From Table 4, it is seen that discriminant validity issues were observed for “doctor services” (in relation to “information”); “organization” (in relation to “doctor services”, “nurse services”, and “information”); and “standard” (in relation to “organization”). This means that these three dimensions were not distinct from the others enough for each to measure the different sub-concepts under patient experience.

### 3.6. Construct Validity, Item Loadings, and Deletion

**Figure 1:**
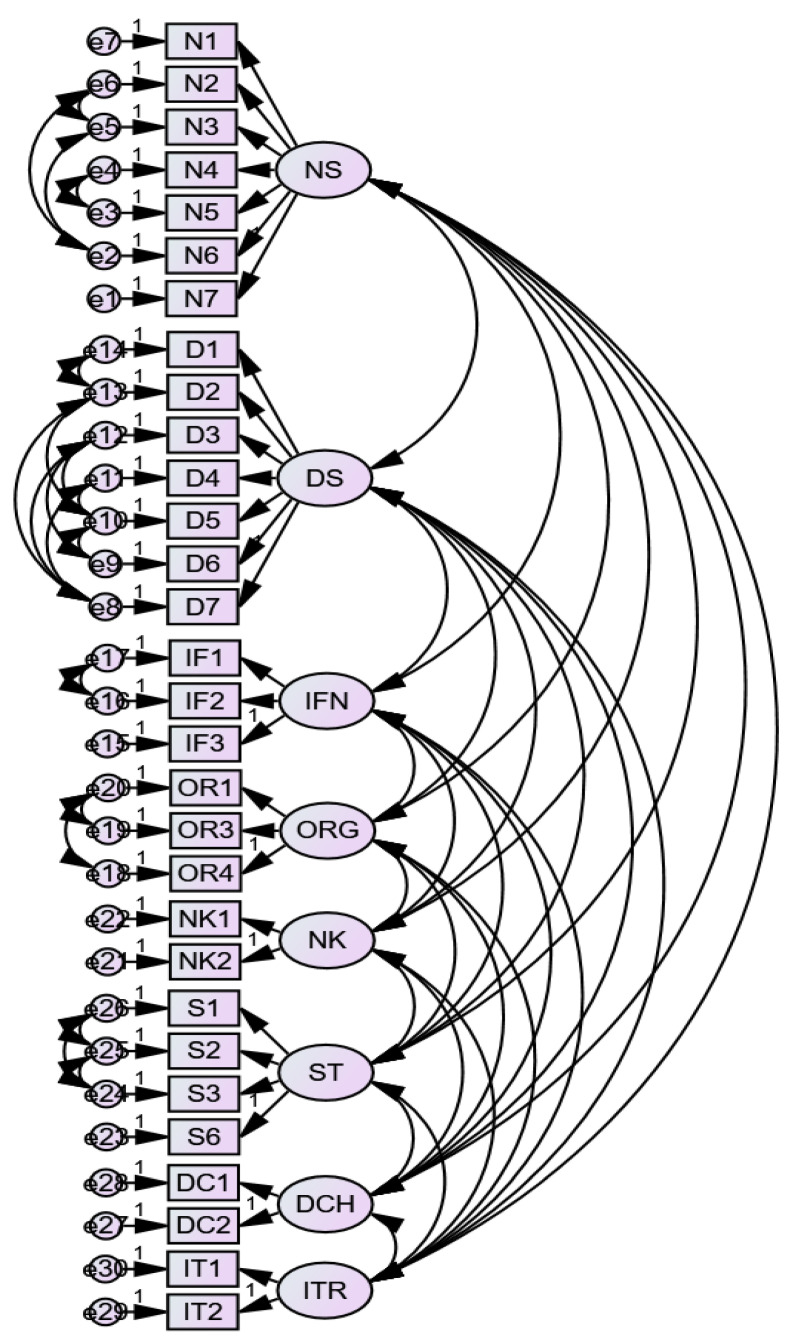
Model after validity checks and item deletion. Note: NS—nurse services; DS—doctor services; INF—information; ORG—organization; ST—standard; NK—next of kin; DCH—discharge; ITR—interaction.

Construct validity for the items was examined by checking item loadings (parameter estimates) on their dimensions. Generally, good loadings were recorded as a majority of the items had loadings above 0.60. The item loadings ranged from 0.88 (on “discharge”) to 0.55 (on “standard”). Two items had loadings below 0.60: 0.58 (ORG 2) and 0.55 (ST 5). Based on the suggestion of the master validity tool [36], these items together with a third (ST4) were deleted in a bid to boost the validity of the measure. Item loadings before and after deletion are presented in Table 5. After deletion, the dimension “standard” recorded an increase in AVE value, indicating that the remaining four items explained more variance in the dimension than the original six items, seen in Table 6. Figure 1 presents the model after item deletion as well as validity and reliability checks. See Model 10 in Table 3 for the fit indices of this model.

### 3.7. Criterion-Related Validity

The study then assessed the predictive validity of the model based on its ability to relate to and predict outcome variables ascertained in existing literature. Overall satisfaction, health benefits, and health level were used as outcome variables while the patient experience measure and its dimensions were used as predicting variables. Patient experience measure and dimensions were computed with retained items after item deletion, and multiple linear regression was performed with age and number of days spent in hospital as control variables. The results showed that overall patient experience and each individual dimension related to and predicted at least one outcome variable positively and significantly. These results are presented in Table 7.

### 3.8. Proposed Measurement Model

**Figure 2:**
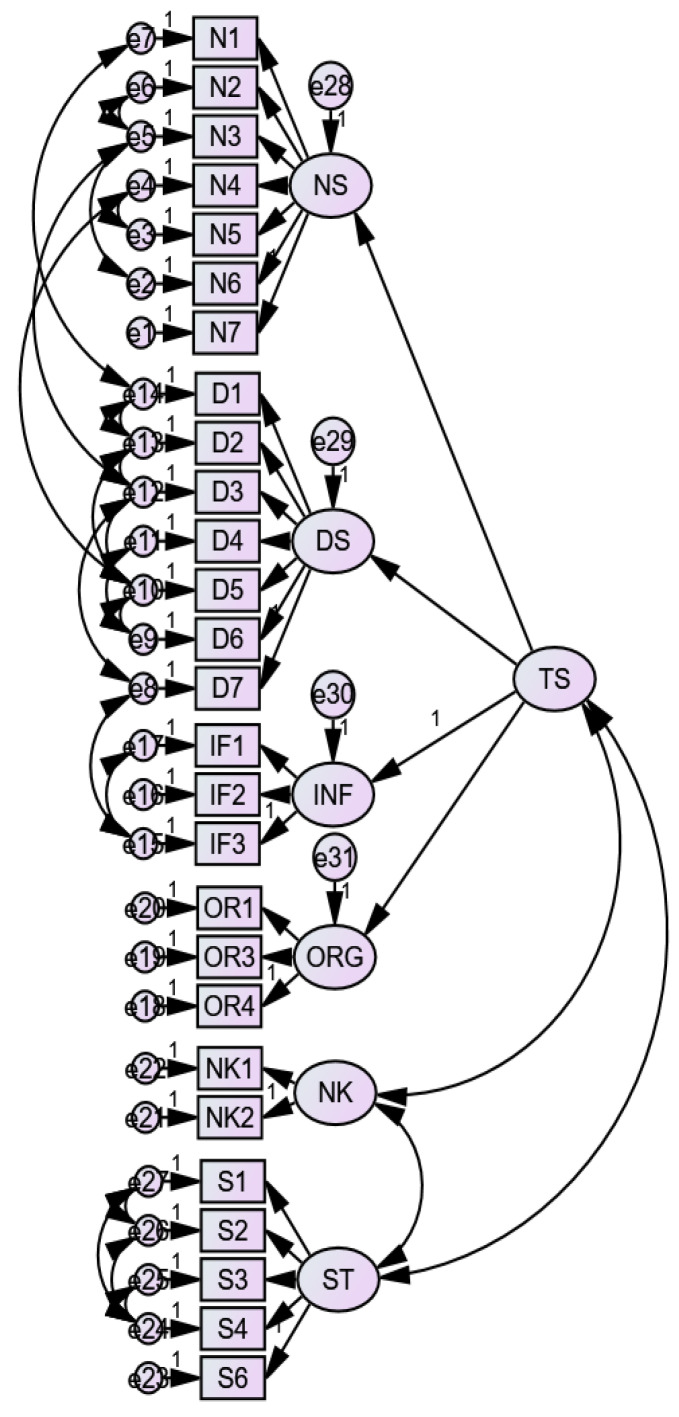
Proposed measurement model. Note: NS—nurse services; DS—doctor services; INF—information; ORG—organization; ST—standard; NK—next of kin; TS—treatment services (second-order factor).

A proposed model (Model 11) was developed, taking into consideration the frequencies of missing values for the items and the discriminant validity concerns. Items with missing values of more than 20% were excluded; therefore, the dimensions of “discharge” and “interaction” were removed from the model. The items on “next of kin” had more than 20% but the dimension was maintained. The questions were the following: “NK1: Were your relatives well received by the hospital staff?” and “NK2: Was it easy for your relatives to get information about you while you were in the hospital?” These questions were maintained because, unlike the other dimensions, relating and answering them depended on factors that are largely beyond the control of the patient, such as whether or not the patient had any relatives alive who visited the hospital and whether the patient stayed in the hospital long enough for relatives to visit the hospital. A second-order factor was added in the proposed model for “nurse services”, “doctor services”, “information”, and “organization”, collectively labelled “treatment services”. This was based on the discriminant validity results, correlations among them, and the nature of the questions asked under these dimensions. The two lowest loading items (ORG 2 and ST 5) that were previously deleted were still excluded from this model. The proposed model showed excellent fitness to the data (similar to Model 10) and also met convergent, discriminant, and criterion-related validity requirements. See Figure 2 for the proposed model. Table 8 presents comparisons of tools and findings between the validation study by Pettersen et al. [8] and the current study.

## 4. Discussion

This study presents some major findings. First, the study confirmed that the eight-factor model showed good fitness to the data. The model achieved configural and metric invariance but not scalar invariance. The study also found that reliability values were all acceptable and all the dimensions, except “standard”, attained the recommended 0.50 AVE value for convergent validity. With regard to discriminant validity, “doctor services” (in relation to “information”), “organization” (in relation to “doctor services”, “nurse services”, and “information”) and “standard” (in relation to “organization”) had issues. Construct validity and criterion-related validity were supported for majority of the results. One item each under “standard” and “organization” had the lowest loadings. Finally, a model including a second-order factor was proposed. The second-order factor, named “treatment services”, consisted of four first-order factors: “nurse services”, “doctor services”, “information”, and “organization”. Moreover, the dimensions of “standard” and “next of kin” were included in this final model, but “discharge” and “interaction” were excluded. Hence, the final model included one second-order factor comprising four sub-factors as well as “standard” and “next of kin”.

The dimensions with associated items found in this study were similar to those found by Pettersen et al. [8] while some dimensions, such as “doctor services”, “nurse services”, “organization”, “information”, and “hospital standards”, overlapped with dimensions found by other studies [5,18,23]. Invariance tests conducted in the present study were absent in the study by Pettersen et al. [8], which marks a good contribution of this study. The tests showed that the model achieved invariance across the aggregated departments with regard to structure and pattern (configural) as well as the loadings of the items on their respective dimensions (metric). However, scalar invariance was not achieved for this model. Considering the diverse nature of the sample, as well as the aggregation of the departments into broad categories, this finding was expected. Putnick and Bornstein [37] asserted that scalar invariance is the most stringent compared with configural and metric, and instances of rigid scalar non-invariance could mean that the construct is generally variant across different groups. The findings also showed that reliability was good, based on composite reliability values, similar to the Cronbach’s alpha values obtained byPettersen et al. [8].

With regard to validity tests, the study found that all the dimensions, except “standard”, attained the recommended 0.50 AVE value for convergent validity, similar to other related studies that examined similar dimensions using other instruments [4]. However, discriminant validity issues were found for “doctor services” (in relation to “information”), “organization” (in relation to “doctor services”, “nurse services”, and “information”) and “standard” (in relation to “organization”). Discriminant validity was also missing in the study by Pettersen et al. [8], thus indicating another good contribution of this study. Examining the wordings of their items gives some possible explanation for this finding. For instance, D1 under “doctor services” was worded as “Did the doctors talk to you so you understood them?”, while questions under “information” included “IF2. Did you know what you thought was necessary about the results of tests and examinations?” and “IF3. Did you receive sufficient information about your diagnosis or your complaints?” It is highly likely that patients will receive information on results and diagnosis mainly from their doctors and, as such, answering questions under “information” may be significantly influenced by the perception of how well the doctors spoke to these patients. Similarly, questions under “organization” were “OR1. Did you find that there was a permanent group of nursing staff that took care of you?”, “OR2. Did you find that one doctor had the main responsibility for you?”, “OR3. Did you find that the hospital’s work was well organized?”, and “OR4. Did you find that important information about you had come to the right person?” These questions feature clear wording relating to “nurse services”, “doctor services”, “information”, and “standard”, and it is therefore not surprising that no clear distinctions were found among them as constructs. Construct validity was also achieved with a majority of the items recording loadings of above 0.60. This was also achieved in the validation study by Pettersen et al. [8] using a different method and in related studies using other instruments with similar dimensions [5,18]. One item on “standard” and one on “organization” were, however, deleted due to loadings below 0.60, while another on “standard” was deleted in a bid to improve the discriminant validity. Perhaps the wording of these questions made them difficult for patients to understand clearly and respond accordingly. For instance, item S5 was framed as “Was the food satisfactory?” Patients may be left to decide what is meant by “satisfactory”, thus making the question too vague, or perhaps the different dietary requirements and preferences made this question more loosely defined. Again, item OR2 was framed as “Did you find that one doctor had the main responsibility for you?”, a question probably dependent on the ailments of the patient and likely to be out of the control of hospital organization. Thus, if a patient’s ailments require more than a single main doctor, then this question may suggest to the patient that having two or more main doctors reduces the ability of the hospitals to organize their work well. Criterion-related validity was ascertained for the overall measure as well as the dimensions in predicting at least one of the three outcome variables: satisfaction, health benefits, and health level, which is consistent with previous studies [2,38,39,40].

Lastly, a model with a second-order factor, “treatment services”, for four of the dimensions was proposed based on the results of the validity and reliability analyses: “nurse services”, “doctor services”, “information”, and “organization”. This constitutes the most important contribution of this study since this possibility was not explored in the study by Pettersen et al. [8], perhaps owing to the absence of discriminant validity examinations in their study, and since this indicates a change in the factor structure of the PEQ. Rindskopf and Rose [41] observed that second-order factors reflect relationships among first-order factors. It is worth noting that related studies that developed other PREMs for generic and specific health issues also found these four dimensions in common [5,17,23]. Although these studies did not develop a second-order factor for these dimensions, this is indicative of the prominence of these four variables in measuring and understanding patient experiences. The current finding, therefore, builds on this prominence to illustrate the high interrelationships and inextricable links among these factors, which brings some theoretical and practical implications to the fore.

### 4.1. Theoretical Implications

This study brings a very important, yet mostly ignored, contribution to the patient experience and quality healthcare literature: a need for more validation studies and surveys on patient experiences. The study responds to the recommendation by Pettersen et al. [8] that existing PREMs require scrutiny and also tackles the research gap identified in the matrix by Beattie et al. [19], indicating that the PEQ by Pettersen et al. [8] lacked some validity analyses. This buttresses the claim that, indeed, changing statistical methods and tools can reveal weaknesses of measures; moreover, this should be countered by regular psychometric appraisals of these measures. The results also contribute to the views of some researchers [20,21], regarding the need to repeat patient experience surveys to generate more reliable data for policy-making. The assessment of patients’ perspectives of hospital care would have to be reliable and valid enough in order to elicit accurate information about their experiences, constructs, and outcomes. Thus, it is imperative to ensure that these instruments always perform optimally and generate reliable information on how to improve quality of care and hospital experiences. These results, therefore, provide a background for further studies to be conducted on PREMs.

Another major contribution of this study is the finding of a second-order factor labelled “treatment services”, which consists of four factors: “nurse services”, “doctor services”, “information”, and “organization”. This means that there exist strong and significant relationships among these dimensions [41]. This finding also means that a single dimension or factor could adequately account for all four dimensions and could be identified as a major sub-dimension that captures these four dimensions. The “treatment services” factor has implications for the conceptualization of patient-oriented hospital service climates. Patients in these hospitals may have highly overlapping experiences across “nurse services”, “doctor services”, “organization”, and “information”. In more specific terms, it can be said that these patients experience a main dimension that accounts for significant portions of the four dimensions, perhaps because of the way these factors play out in the hospitals. For instance, doctors provide information regarding patients’ health, ailments, and treatments while nurses organize and assist patients with the treatment process. This is significant in advancing knowledge of patient experiences. The experience of these four dimensions may not be that distinct, and patients, in experiencing service climate in the hospitals, may not adequately distinguish their shared perceptions of “doctor services” from “information” or of “nurse services” from “organization”, for instance. The climate in the hospitals during healthcare delivery may thus be experienced and perceived by patients as having two levels of factors. This contribution is also a major highlight when compared with the study by Pettersen et al. [8], in which discriminant validity was not examined and a resulting second-order factor analysis was not explored. This challenges the theoretical structure of the PEQ and theoretical distinctness among these factors. Therefore, this study suggests a change in the factor structure of the PEQ and the development of a second-order factor for these four dimensions in the general patient experience literature. These possibilities are worth exploring in further surveys and studies on hospital factors as patient experiences during the caregiving process.

### 4.2. Practical Implications

Quality healthcare delivery is not exclusive to a region or country but a general goal of all healthcare systems worldwide. This can be contributed to by generating accurate information on how healthcare users experience healthcare systems. The results from this study suggest that it is not enough to develop a good measure of patient experiences, but it is imperative to review and reassess the ability of the measure to keep generating accurate information on patients’ experiences and health. The questions in the PEQ may have to be revised in order to elicit more concise and accurate information from patients. Furthermore, some dimensions, such as “next of kin”, seemed not to be relatable to most of the patients, judging from the many missing values and invariance tests. In addition, the PEQ should be administered with the second-order factor taken into consideration. It is imperative to analyse “nurse services”, “doctor services”, “information”, and “organization” as a second-order factor, as shown in the proposed model, due to the validity issues that were realized in the analysis. This can provide researchers and management with adequate knowledge on what patients experience during the caregiving process. Moreover, management must take the interrelationships in the second-order factor into account to make meaningful, informed, and sustainable changes in the hospitals for patients. The second-order factor must be considered as a single factor encompassing these four dimensions, where patients’ perceptions and interactions with a dimension have a ripple effect on the others. Such considerations in policies and practice can help management and workers to reduce errors that may have dire consequences.

### 4.3. Limitations and Directions for Future Research

This study employs data that is not at the national level but from a health region in Norway. That notwithstanding, the study has good generalizability power owing to the similarity in hospital and healthcare systems across the regions in Norway. Generalizing to other countries, however, is difficult due to the differences in culture and healthcare systems. The findings require additional research in different countries for further justification. Therefore, future studies on reassessing psychometric properties of PREMs may want to employ larger data sets, for instance at the national level or across regions, to further investigate and develop the measurement quality of such surveys. Furthermore, future research should adopt the proposed model (with the second-order factor) from this study and examine it empirically to confirm it or otherwise, within health sectors across different countries. It is also worth noting that only nurses’ and doctors’ services were assessed but not the services of other healthcare professionals in hospitals. Future research on developing and improving PREMs should therefore incorporate questions that assess the experience of services of other professionals.

## 5. Conclusions

Hospital management should know and consider the views and experiences of the people they care for if their services are to be influential in improving patients’ health. The results of this study show that changes in psychometric analytical tools and methods can indeed highlight possible weaknesses and inadequacies in measures, as seen with the PEQ. This is evident in analyses such as invariance, discriminant validity, and second-order factors conducted in the current study but absent in the earlier study. Therefore, repeated surveys with refined and further developed questionnaires are needed to hopefully improve the performance of the measures. The results also indicate possible changes with regard to dimensionality of PREMs, owing to the second-order factor finding. This calls for adequate attention, from researchers and hospital management alike, to the interrelationships among some of the dimensions, as this has important implications for theory and practice in healthcare. Management should consider these relationships in making decisions concerning the quality of care for patients, while researchers should delve more into studies that ascertain the psychometrics and dimensionality of PREMs.

## Figures and Tables

**Table 1:** Fitness indices and acceptable thresholds.

Fit Indices	Acceptable Thresholds
CFI	>0.95, excellent; >0.90, acceptable
TLI	>0.95, excellent; >0.90, acceptable
RMSEA	<0.06, excellent; 0.06–0.10, moderate
PCLOSE	>0.05, excellent

Adapted from Hu and Bentler (1999).

**Table 2:** Sample characteristics.

Variables	Frequency	Valid Percent
Age		
Less than 61 years	1502	32.6
61–73 years	1528	33.2
73 years and above	1573	34.2
Days spent in hospital		
Less than 4 days	2630	57.1
4 or more days	1973	42.9
Department aggregates		
Medical (Med)	2468	53.6
Surgical (Kir)	2135	46.4
Hospitals		
Hospital 1	2067	44.9
Hospital 2	1084	23.5
Hospital 3	193	4.2
Hospital 4	794	17.2
Hospital 5	465	10.1

**Table 3:** Fitness results for all models.

Fit Indices	Model 1—Initial Model without Modifications	Model 2 *—Model after Modifications	Models 3–8 *—Alternative Models	Model 9 *—Configural Invariance	Model 10 *—Model after Item Deletion	Model 11 *—Proposed Model
			1st 2nd 3rd 4th 5th 6th			
CFI	0.91	0.95	0.91 0.91 0.92 0.88 0.86 0.85	0.95	0.95	0.96
TLI	0.89	0.94	0.89 0.89 0.90 0.85 0.84 0.82	0.94	0.94	0.95
RMSEA	0.06	0.04	0.06 0.06 0.05 0.07 0.07 0.07	0.03	0.04	0.04
PCLOSE	0.00	1.00	0.00 0.00 0.00 0.00 0.00 0.00	1.00	0.99	0.98

Note: * These models were assessed with the modification estimates. 1st—nurse and doctor into one factor; 2nd—nurse, doctor and organization into one factor; 3rd—nurse and organization into one factor; doctor and information into one factor; 4th—nurse, doctor, organization, and information into one factor; next of kin and standard into one factor; discharge and interaction into one factor; 5th—nurse, doctor, organization, information, next of kin, and standard into one factor; discharge and interaction into one factor; 6th—all dimensions into one factor.

**Table 4:** Correlations, reliability, convergent, and discriminant validity before item deletion (Model 2).

	CR	AVE	1	2	3	4	5	6	7	8
1. Nurse services	0.90	0.57	**0.76**							
2. Doctor services	0.92	0.64	0.80	**0.80**						
3. Information	0.87	0.70	0.77	0.82	**0.83**					
4. Organization	0.81	0.53	0.85	0.80	0.79	**0.73**				
5. Next of kin	0.83	0.70	0.69	0.59	0.63	0.71	**0.84**			
6. Standard	0.82	0.44	0.65	0.57	0.56	0.73	0.60	**0.67**		
7. Discharge	0.87	0.77	0.57	0.56	0.63	0.58	0.49	0.44	**0.88**	
8. Interaction	0.72	0.57	0.57	0.58	0.56	0.63	0.53	0.50	0.56	**0.75**

Note: CR—composite reliability; AVE—average variance explained; figures in bold are the square roots of the AVEs for discriminant validity (using the Fornell–Larcker procedure; discriminant validity is supported when the square root of the AVEs are greater than the correlation coefficients between the constructs).

**Table 5:** Standardized factor loadings (before and after item deletion) and missing values.

Dimensions and Items	Factor Loadings			
	Model 2	Model 10	Model 11	Missing Values *N* (%)
Nurse services				
N1. Did the nursing staff talk to you so you understood them?	0.67	0.66	0.67	287 (6.2)
N2. Did you find that the nursing staff cared for you?	0.80	0.80	0.79	293 (6.4)
N3. Do you have confidence in the professional skills of the nursing staff?	0.78	0.78	0.78	282 (6.2)
N4. Did you tell the nursing staff everything you thought was important about your condition?	0.72	0.72	0.72	340 (7.4)
N5. Did you find that the nursing staff were interested in your description of your own situation?	0.83	0.83	0.83	328 (7.1)
N6. Were you included in the advice on questions regarding your care?	0.70	0.70	0.68	427 (9.3)
N7. Did the nursing staff have time for you when you needed it?	0.77	0.77	0.78	297 (6.5)
Doctor services				
D1. Did the doctors talk to you so you understood them?	0.73	0.73	0.73	300 (6.5)
D2. Did you find that the doctors took care of you?	0.84	0.84	0.83	302 (6.6)
D3. Do you trust the doctors’ professional skills?	0.77	0.77	0.77	299 (6.5)
D4. Did the doctors have time for you when you needed it?	0.82	0.82	0.82	415 (9.0)
D5. Did you tell the doctors everything you thought was important about your condition?	0.77	0.77	0.77	384 (8.3)
D6. Did you find that the doctors were interested in your description of your own situation?	0.84	0.84	0.85	378 (8.2)
D7. Did you find that the treatment was adapted to your situation?	0.79	0.79	0.76	321 (7.0)
Information				
IF1. Did you know what you thought was necessary about how tests and examinations should take place?	0.79	0.79	0.85	320 (7.0)
IF2. Did you know what you thought was necessary about the results of tests and examinations?	0.85	0.85	0.86	334 (7.3)
IF3. Did you receive sufficient information about your diagnosis or your complaints?	0.86	0.86	0.87	326 (7.1)
Organization				
OR1. Did you find that there was a permanent group of nursing staff that took care of you?	0.67	0.67	0.68	121 (2.6)
* OR2. Did you find that one doctor had the main responsibility for you?	0.58	-	-	130 (2.8)
OR3. Did you find that the hospital’s work was well organized?	0.81	0.82	0.82	107 (2.3)
OR4. Did you find that important information about you had come to the right person?	0.82	0.81	0.81	204 (4.4)
Next of kin				
NK1. Were your relatives well received by the hospital staff?	0.84	0.84	0.84	1362 (29.6)
NK2. Was it easy for your relatives to get information about you while you were in the hospital? Standard	0.83	0.83	0.83	1732 (37.6)
S1. Did you get the impression that the hospital equipment was in good condition?	0.71	0.72	0.78	108 (2.3)
S2. Did you get the impression that the hospital was in good condition?	0.77	0.78	0.86	122 (2.7)
S3. Was the room you were in satisfactory?	0.67	0.65	0.74	80 (1.7)
* S4. Was the opportunity for rest and rest satisfactory?	0.62	-	0.66	90 (2.0)
* S5. Was the food satisfactory?	0.55	-	-	122 (2.7)
S6. Was the cleaning satisfactory?	0.65	0.66	0.60	89 (1.9)
Discharge				
DC.1 Were you informed of what you could do at home in case of relapse?	0.87	0.87	-	1327 (28.8)
DC2. Were you informed of what complaints you could expect to receive in time after your hospital stay?	0.88	0.88	-	1195 (26.0)
Interaction				
IT1. Do you find that the hospital has worked well with your GP about what you were admitted to?	0.82	0.81	-	2523 (33.9)
IT2. Do you feel that the hospital has cooperated well with the home or other municipal services about what you were admitted for?	0.69	0.69	-	3401 (54.8)
Treatment services				
Nurse services			0.92	
Doctor services			0.86	
Information			0.84	
Organization			0.93	

Note: Items marked with * had the lowest loadings.

**Table 6:** Correlations, reliability, convergent, and discriminant validity after item deletion (Model 10).

	CR	AVE	1	2	3	4	5	6	7	8
1. Nurse services	0.90	0.57	**0.76**							
2. Doctor services	0.92	0.64	0.80	**0.80**						
3. Information	0.87	0.70	0.77	0.82	**0.83**					
4. Organization	0.81	0.59	0.87	0.78	0.79	**0.77**				
5. Next of kin	0.82	0.70	0.69	0.59	0.63	0.72	**0.84**			
6. Standard	0.80	0.50	0.64	0.56	0.55	0.74	0.59	**0.70**		
7. Discharge	0.87	0.77	0.57	0.56	0.63	0.56	0.49	0.44	**0.88**	
8. Interaction	0.72	0.57	0.57	0.58	0.56	0.62	0.53	0.47	0.56	**0.75**

Note: CR—composite reliability; AVE—average variance explained; figures in bold are the square roots of the AVEs for discriminant validity (using the Fornell–Larcker procedure; discriminant validity is supported when the square root of the AVEs are greater than the correlation coefficients between the constructs).

**Table 7:** Regression results for criterion-related validity.

Outcome Variables							
Model 10				Proposed Model (Model 11)			
	Satisfaction	Health Benefits	Health Level		Satisfaction	Health Benefits	Health Level
Predictors				Predictors			
Overall patient experience	0.52 ***	0.47 ***	0.19 ***	Treatment services	0.57 ***	0.50 ***	0.28 ***
Nurse services	0.35 ***	0.18 ***	0.10 ***	Standard	0.20 ***	0.10 ***	0.00
Doctor services	0.07 ***	0.12 ***	0.10 ***	Next of kin	0.02	0.01	−0.07 ***
Information		0.09 ***					
Organization	0.19 ***	0.10 ***					
Next of kin			0.07 ***				
Standard	0.17 ***	0.08 ***					
Discharge		0.09 ***	0.13 **				

*** *p* < 0.001; ** *p* < 0.01; empty fields are not significant at 0.05 level; Treatment services—second order factor comprising nurse services, doctor services, information, and organization.

**Table 8:** Tools and findings in the earlier validation study and the current study.

Study	Psychometric Tools Used	Findings
Pettersen et al. (2004)	Exploratory factor analysis	10 factors (including general satisfaction)
	Cronbach’s alpha test	Confirmed
	Test-retest reliability	Confirmed
	Construct validity	Achieved
Current study	Confirmatory factor analysis	8 factors (excluding general satisfaction)
	Model comparisons	Initial model was found to be best
	Measurement invariance	Configural and Metric achieved, Scalar not achieved
	Composite reliability test	Confirmed
	Convergent validity	Confirmed for all except one factor
	Discriminant validity	Confirmed for all except three factors
	Construct validity	Achieved
	Criterion-related validity	Achieved
	Second-order factor analysis	Achieved composite reliability, convergent validity, discriminant validity, construct validity and criterion related validity

## Data Availability

The data presented in this study are available on request from the corresponding author. The data are not publicly available due to an ongoing research project.

## Supplementary Materials

The following are available online at https://www.mdpi.com/article/10.3390/ijerph18137141/s1, Table S1. Metric invariance across department aggregates; Table S2. Scalar invariance across department aggregates.
