# Supplementary material for: Validation and Adjustment of the Patient Experience Questionnaire (PEQ): A Regional Hospital Study in Norway
Source: Int J Environ Res Public Health. 2021 Jul 3;18(13):7141. doi: 10.3390/ijerph18137141 (PMC8296920; doi:10.3390/ijerph18137141)
Supplement: Supplementary file 1 [file ijerph-18-07141-s001.zip › ijerph-1247630-supplementary.pdf]

Table S1. Metric invariance across department aggregates.

Item		Dim.	Kir		Med		z-Stat
			Estimate	*p*	Estimate	*p*	
**sp9**	<---	N6	1.23	0.00	1.26	0.00	0.42
**sp8**	<---	N5	1.13	0.00	1.15	0.00	0.43
**sp7**	<---	N4	0.92	0.00	0.99	0.00	1.77 *
**sp6**	<---	N3	0.87	0.00	0.90	0.00	0.86
**sp5**	<---	N2	0.91	0.00	0.93	0.00	0.76
**sp4**	<---	N1	0.65	0.00	0.75	0.00	3.43 ***
**sp16**	<---	D6	1.27	0.00	1.16	0.00	−2.56 **
**sp15**	<---	D5	1.15	0.00	1.07	0.00	−1.97 **
**sp14**	<---	D4	1.25	0.00	1.12	0.00	−3.06 ***
**sp13**	<---	D3	0.82	0.00	0.88	0.00	1.81 *
**sp12**	<---	D2	1.15	0.00	1.07	0.00	−2.08 **
**sp11**	<---	D1	0.90	0.00	0.85	0.00	−1.57
**sp19**	<---	IF3	0.98	0.00	0.99	0.00	0.34
**sp18**	<---	IF1	0.84	0.00	0.85	0.00	0.19
**sp24**	<---	OR3	0.93	0.00	0.95	0.00	0.50
**sp23**	<---	OR2	0.97	0.00	0.90	0.00	−1.34
**sp22**	<---	OR1	0.87	0.00	0.89	0.00	0.58
**sp26**	<---	NK1	0.95	0.00	0.84	0.00	−2.56 **
**sp32**	<---	ST5	0.85	0.00	0.83	0.00	−0.31
**sp31**	<---	ST4	1.06	0.00	1.07	0.00	0.01
**sp30**	<---	ST3	0.97	0.00	1.03	0.00	0.91
**sp29**	<---	ST2	0.91	0.00	1.00	0.00	1.73 *
**sp28**	<---	ST1	0.89	0.00	0.98	0.00	1.67 *
**sp44**	<---	DC1	1.00	0.00	0.97	0.00	−0.69
**sp47**	<---	IT1	1.11	0.00	1.18	0.00	0.55

Note: * ($p < 0.05$), ** ($p < 0.01$), *** ($p < 0.001$)—not metrically invariant; NS: nurse services; DS: doctor services; IFN: information; ORG: organisation; NK: next of kin; ST: standard; DCH: discharge; ITR: interaction.

Table S2. Scalar invariance across department aggregates.

Model	DF	CMIN	P
Measurement weights	23	61.20	0.00
Measurement intercepts	23	61.20	0.00
Structural covariances	73	254.70	0.00
Measurement residuals	119	391.29	0.00

Note: $p < 0.05$- no scalar invariance.
